# Die Auswirkungen der Frühphase der COVID-19 Pandemie auf die Erwerbssituation und die finanzielle Lage von Familien in Österreich

**DOI:** 10.1007/s11614-021-00466-9

**Published:** 2021-11-30

**Authors:** Nadia Steiber, Christina Siegert

**Affiliations:** 1 Institut für Soziologie, Universität Wien, Rooseveltplatz 2, 1090 Wien, Österreich; 2 Institut für Höhere Studien, Wien, Österreich

**Keywords:** Corona-Krise, COVID-19, Familie, Haushaltseinkommen, Arbeitsmarkt, Armut, Corona crisis, COVID-19, Family, Household income, Labour market, Poverty

## Abstract

In dieser Forschungsnotiz werden erste Ergebnisse der AKCOVID-Studie vorgestellt. Diese untersucht auf Basis von repräsentativen Befragungsdaten die Auswirkungen der Pandemie auf die finanzielle Lage von Familien in Österreich. Dabei wird die Situation von Haushalten mit Kindern vor Beginn der Corona-Krise (Februar 2020) mit der Situation im Frühsommer 2020 verglichen. Die deskriptiven Ergebnisse zeigen, dass ein großer Teil der Familien bereits drei Monate nach Beginn der Krise die ökonomischen Folgen der Pandemie spürte und aufgrund krisenbedingter Veränderungen der elterlichen Erwerbssituation auf Teile des Haushaltseinkommens verzichten musste. Die Zahl der Familien mit finanziellen Problemen (subjektive Armutsgefährdung) stieg deutlich an, v. a. unter Alleinerziehenden und Paaren mit mehr als zwei Kindern. Damit wird deutlich, dass vor allem jene Familien, die sich bereits vor der Krise in einer vulnerablen finanziellen Situation befanden, schon sehr früh direkt von den ökonomischen Folgen der Pandemie betroffen waren. Viele Familien sorgten sich, dass sie aufgrund der Krise Einkommenseinbußen erleiden und finanzielle Probleme bekommen werden.

## Abstract (en)

This research note presents first results of the AKCOVID study. Based on representative survey data the study examines the impact of the pandemic on the economic situation of families with children in Austria. We compared the situation of couples with dependent children and single parents before the start of the crisis (February 2020) with the situation in June 2020. The descriptive results indicate that a large proportion of families were already affected by the economic consequences of the pandemic three months after the onset of the Corona crisis. Pandemic-related changes in the employment situation of parents led to significant financial losses and rising shares of families with difficulties managing on their current household income (*subjective* poverty risk). Among the most strongly affected families were couples with more than two minor children and single parents. The analyses illustrate the direct and massive impact of the pandemic on families in Austria. Especially those who were already in a vulnerable financial situation before the crisis felt its economic impact. And many of those who were not yet affected worried that they may still run into financial problems in the further course of the pandemic.

## Einleitung

Insbesondere für Familien mit Kindern stellt die COVID-19-Krise eine große Belastung dar. Das zeitweise Wegbrechen institutioneller und informeller Kinderbetreuungsangebote, die Umstellung auf *Distance Learning* an Schulen sowie die Verwerfungen am Arbeitsmarkt wirkten sich negativ auf die zeitlichen und auch emotionalen Ressourcen der Eltern und das Wohlbefinden der Kinder aus (Berghammer und Beham-Rabanser 2020; Schönherr 2020; Steiber 2021b; Zartler 2020). Im Fokus dieser Forschungsnotiz stehen die krisenbedingten Veränderungen in der elterlichen Erwerbssituation und der finanziellen Lage von Familien mit Kindern in Österreich, die sich bereits drei Monate nach Beginn der Krise zeigten.

Um dem raschen Anstieg der Arbeitslosigkeit zu Beginn der Krise entgegenzuwirken, handelten die Sozialpartner ein Kurzarbeitsmodell aus, das sehr intensiv genutzt wurde. In der Hochphase im April 2020 waren etwa 30 % der Beschäftigten in Österreich in Kurzarbeit (Huemer et al. 2021). Dadurch konnten krisenbedingte Arbeitsplatzverluste eingedämmt werden: Im Juni 2020 waren etwa 4 % der im Februar 2020 unselbstständig Beschäftigten ohne Job (Steiber et al. 2021a, S. 5). Diese Entwicklungen hatten direkte Auswirkungen auf die Einkommenssituation der privaten Haushalte. Im Jahr 2020 erhielten unselbstständig Beschäftigte in „Corona-Kurzarbeit“ zwischen 80 und 90 % ihres letzten Nettoeinkommens. Trotz der hohen Lohnersatzraten kann Kurzarbeit für Niedriglohnbezieher*innen existenzbedrohend sein. Die Berechnungsgrundlage für das Kurzarbeitsgeld ist das vertragliche Bruttoeinkommen. Damit entfallen Einkommenskomponenten wie Trinkgeld und Überstundenentgelt, die in vielen Bereichen elementare Bestandteile des Nettoeinkommens sind (Theurl 2020). Auch die pandemiebedingt gestiegene Zahl an arbeitslosen Menschen und die Umsatzeinbußen vieler Unternehmer*innen hatten einen direkten Einfluss auf die finanzielle Situation privater Haushalte. Bei Leistungsanspruch beziehen Arbeitslose als Grundbetrag 55 % des letzten Nettoeinkommens und Zuschläge für finanziell abhängige Familienmitglieder.^1^ Zur Abfederung der krisenbedingten Einkommensverluste wurden Einmalzahlungen an Arbeitslose geleistet und Härtefallfonds für Selbstständige und für Familien mit Kindern geschaffen (Heitzmann 2020).

Bisher wurden die Effekte der Pandemie auf die Einkommen privater Haushalte in Österreich v. a. auf Basis von Simulationsstudien diskutiert (u. a. bei Fink et al. 2020; Albacete et al. 2021). Diese Studien basieren auf Strukturdaten zu Haushalten und Arbeitsmarktrisiken sowie auf Informationen zur sozialrechtlichen Ausgestaltung des Abgaben- und Sozialleistungssystems und der Krisenmaßnahmen – nicht auf aktuellen Daten zur tatsächlichen Einkommenssituation der Haushalte. Laut Simulationsergebnissen konnten einkommensschwache Haushalte, deren Einkommen sich stärker aus Pensionen, Transfer- und Sozialleistungen speisen, ihr Einkommensniveau im ersten Krisenjahr eher halten als einkommensstärkere Haushalte (zu einem ähnlichen Schluss kommen Almeida et al. 2020 und Bruckmeier et al. 2020 für Deutschland). Die Studien verweisen jedoch auch auf die ausgeprägte finanzielle Vulnerabilität bestimmter Haushaltskonstellationen: Alleinerziehende und Familien mit drei oder mehr Kindern haben häufig wenig finanziellen Spielraum (d. h. ein geringes verfügbares Nettoeinkommen nach Abzug der Lebenserhaltungskosten und oft keine Ersparnisse) und können Einkommenseinbußen daher besonders schlecht verkraften (Albacete et al. 2021). Das Risiko, durch Einkommensverluste zu verarmen, ist insbesondere bei Alleinerziehenden aufgrund des Fehlens einer weiteren Erwerbsperson im Haushalt stark erhöht (Goerne 2011). Bereits vor Beginn der Krise war etwa ein Drittel der Alleinerziehenden armutsgefährdet.^2^ Das Armutsrisiko für Familien mit drei oder mehr Kindern war mit 31 % fast drei Mal so hoch wie für Familien mit einem oder zwei Kind/ern (Statistik Austria 2021a, S. 166). Es steht zu befürchten, dass sich der Anteil der armutsgefährdeten Familien durch die Corona-Krise erhöht hat (Dawid 2020; Heitzmann 2020). Obgleich sich die nachhaltigen Auswirkungen der Pandemie auf die Armutsgefährdung in Österreich erst in den nächsten Jahren manifestieren werden, ist es wichtig, bereits die frühen und, wie zu befürchten steht, nicht nur kurzlebigen Auswirkungen der COVID-19-Krise auf die finanzielle Lage der Familien zu untersuchen.

Die Administrativdaten des Hauptverbands der Sozialversicherungsträger und des Arbeitsmarktservice (AMDB) stehen zeitnah für die Wissenschaft zur Verfügung, bilden jedoch keine Haushaltszusammenhänge ab. Offizielle Zahlen zur finanziellen Lage und Armutsgefährdung von Privathaushalten im Pandemiejahr 2020 werden aller Voraussicht nach erst im Jahr 2022 zur Verfügung stehen.^3^ Vor diesem Hintergrund war ein Ziel der AKCOVID-Studie, die frühen Krisenfolgen für Familien auf Basis von Befragungsdaten abzubilden. Wir untersuchen in dieser Forschungsnotiz auf Basis von Befragungsdaten für Juni 2020, wie groß der Anteil der Familien mit Kindern war, in denen drei Monate nach Beginn der Krise zumindest ein Elternteil entweder den Arbeitsplatz verloren hat oder zu Kurzarbeit angemeldet war, und welche Auswirkungen diese Verwerfungen am Arbeitsmarkt für die finanzielle Lage der Familien hatten. Wie wirkten sich die pandemiebedingte Wirtschaftskrise bzw. deren erhoffte Abfederung durch staatliche Krisenmaßnahmen auf die *von Familien erlebte* finanzielle Lage aus?

Der Beitrag gliedert sich in drei weitere Abschnitte. Abschn. 2 beschreibt das Design der AKCOVID-Studie, die Pandemie- und Arbeitsmarktsituation zum Zeitpunkt der Befragung und die Befragungsdaten. Abschn. 3 gibt einen kurzen Überblick über die deskriptiven Studienergebnisse. Dabei werden zuerst die finanziellen Folgen von Kurzarbeit und Arbeitslosigkeit für Paarhaushalte mit Kindern und Alleinerziehende umrissen. Dann werden die daraus resultierenden finanziellen Zukunftssorgen nach Haushaltstypus und sozialem Status analysiert. Im vierten Abschnitt werden die Ergebnisse zusammengefasst und im Hinblick auf aktuelle Krisenentwicklungen reflektiert.

## Daten und Methoden

Die Basis der Studie bildet eine repräsentative Befragung von 2000 in Österreich lebenden Personen im Alter von 20 bis 64 Jahren (AKCOVID 2021; Steiber 2021a), die im Juni 2020^4^ von IFES durchgeführt wurde. Die Daten wurden in einer Zeit erhoben, in der die Pandemie temporär unter Kontrolle war (niedrige Infektionszahlen) und sich die Arbeitsmarktlage entspannte. Die Zahl der vorgemerkten Arbeitslosen (inkl. Schulungsteilnehmer*innen) fiel während des zweiwöchigen Befragungszeitraums von rund 476 Tausend auf rund 447 Tausend Personen (AMDB 2021). Gleichzeitig waren im Juni 2020 nach wie vor mehr als eine halbe Million Personen (AMDB 2021) bzw. rund 15 % der in Februar 2020 noch regulär unselbstständig Erwerbstätigen für Kurzarbeit angemeldet (Steiber et al. 2021a, b).

Die AKCOVID-Studie zielte auf eine Überrepräsentation von *Haushalten mit minderjährigen Kindern,* um eine detaillierte Analyse unterschiedlicher Familienkonstellationen zu erlauben. Als* Familien* definieren wir alle Eltern-Kind-Gemeinschaften, die in einem Haushalt leben. Der Schwerpunkt der Analyse ist auf elterlichen Haushalten, in denen zumindest ein minderjähriges Kind lebt (leibliche Kinder, Stief‑, Pflege- oder Adoptivkinder). Das Analysesample umfasst 799 in Österreich lebende Elternpaare sowie 106 Alleinerziehende (Tab. 1). Diese können mit 565 kinderlosen Paarhaushalten (ohne Kind im Alter von unter 18 Jahren) verglichen werden. Pro Haushalt wurde eine Person interviewt; die Erwerbssituation der im gemeinsamen Haushalt lebenden Partner*innen wurde stellvertretend abgefragt. Die Daten wurden mittels computerunterstützter Telefon- (20 %) und Web-Interviews (80 %) erhoben. Das verwendete Multi-Mode-Design gewährleistet, dass auch Personen in der Studie repräsentiert sind, die aus diversen Gründen nicht an Online-Interviews teilnehmen können. Die Daten wurden für diesen Beitrag deskriptiv ausgewertet (unter Verwendung eines Poststratifizierungsgewichts, das Verzerrungen im Analysesample in Bezug auf Alter, Bildung, Bundesland und Haushaltskonstellation ausgleicht).

**Table 1:** 

	Gesamt		Familien	
	Anzahl der Beobachtungen	Gewichtete Anteile^c^	Anzahl der Beobachtungen	Gewichtete Anteile^c^
*Geschlecht*				
Frauen	1003	51 %	459	49 %
Männer	997	49 %	446	51 %
*Alter*				
20–29	347	20 %	122	7 %
30–39	492	22 %	369	31 %
40–49	483	23 %	287	36 %
50–59	519	26 %	120	24 %
60–64	159	9 %	7	3 %
*Haushaltstypus*				
Alleinlebende	378	23 %	–	–
Alleinerziehende^a^ mit Kind <18 Jahre	106	4 %	106	10 %
Paare^b^ ohne Kind	565	27 %	–	–
Paare, jüngstes Kind <6 Jahre	392	14 %	392	35 %
Paare, jüngstes Kind 6 < 18 Jahre	407	21 %	407	55 %
Andere	152	11 %	–	–
*Erwerbsstatus Juni 2020*				
Erwerbstätig inkl. Selbstständige	1173	58 %	565	65 %
Kurzarbeit	259	13 %	125	13 %
Arbeitslos/AMS-Schulung	159	9 %	61	7 %
Nicht oder geringfügig erwerbstätig/Karenz	407	20 %	154	15 %
Keine Information	2	0 %	0	0 %
*Gesamt*	*2000*	*100%*	*905*	*100%*

Die Befragungsdaten (GESAMT) umfassen eine repräsentative Stichprobe der in Österreich lebenden Bevölkerung im Alter von 20 bis 64 Jahren. Das Analysesample für die vorliegende Untersuchung (FAMILIEN) bezieht sich auf Paare und Alleinerziehende mit Kind/ern unter 18 Jahren.

^a^Rund 75 % der Alleinerziehenden in der Stichprobe sind Frauen.

^b^Rund 2,2 % der Paare sind gleichgeschlechtlich (*N* = 29).

^c^Post-Stratifikationsgewichtung, die unter anderem für das bewusste Übersampling von Familien mit minderjährigen Kindern korrigiert.

### Erhebung der Erwerbssituation

Der Erwerbsstatus der Respondent*innen (und ihrer Partner*innen) wurde retrospektiv für Februar 2020 und für den Befragungszeitpunkt erhoben. Die Erwerbsstrukturen aus der Befragung decken sich dabei weitgehend mit den Administrativdaten des Hauptverbands der Sozialversicherungsträger und des Arbeitsmarktservice (AMDB): Laut Befragung war jede dritte Person, die im Februar noch regulär unselbstständig erwerbstätig war, innerhalb des ersten Halbjahrs 2020 in Kurzarbeit. Zum Zeitpunkt der Befragung im Juni 2020, waren rund 15 % der im Februar noch regulär unselbstständig Erwerbstätigen in Kurzarbeit und etwa 4 % waren arbeitslos gemeldet. Auch laut der Administrativdaten waren von den rund 3,7 Mio. Personen, die im Februar 2020 über der Geringfügigkeitsgrenze erwerbstätig waren, im Juni 2020 rund 4 % arbeitslos (3,6 % der Männer und 4,2 % der Frauen) sowie rund 15 % in Kurzarbeit (16,1 % der Männer und 14,9 % der Frauen, cf. Steiber et al. 2021a, S. 5). Das weist auf eine hohe Qualität der Befragungsdaten. Beide Datenquellen deuten auf geringe geschlechtsspezifische Unterschiede in Bezug auf das Risiko von krisenbedingten Job- und Einkommensverlusten (Steiber et al. 2021a, S. 5, 2021b, S. 8).

Um die Betroffenheit der Haushalte durch krisenbedingte Veränderungen der Erwerbssituation mit Implikationen für das Einkommen messen zu können, verwenden wir einen ähnlichen Ansatz wie Albacete et al. (2021): Wir schätzen die Betroffenheit der Haushaltsmitglieder von Arbeitslosigkeit *oder* Kurzarbeit im Juni 2020 und fokussieren auf Haushaltsmitglieder, die kurz vor der Krise (Februar 2020) noch über der Geringfügigkeitsgrenze unselbstständig erwerbstätig waren, um die (überwiegend) *krisenbedingten* Veränderungen zu erfassen.

### Erhebung der finanziellen Lage

Die Beurteilung der Einkommenssituation der Haushalte vor bzw. während der Pandemie basiert, in Anlehnung an die Messung im European Social Survey, auf der Fragestellung „*Wie würden Sie die Einkommenssituation Ihres Haushalts beurteilen* –* vor Beginn der Corona-Krise im Februar 2020 im Vergleich zu heute?*“ (1 – *bequem leben*, 2 – *zurechtkommen*, 3 – *schwer zurechtkommen*, 4 – *sehr schwer zurechtkommen*). Finanzielle Notlagen auf Basis von zwei Aussagen und anhand einer fünfteiligen Skala von 1 – *trifft gar nicht zu* über 3 – *teils-teils* bis 5 – *trifft voll und ganz zu* erhoben: „*Ich muss/wir müssen seit Beginn der Corona-Krise auf Ersparnisse zurückgreifen oder Schulden machen, um den normalen Lebensunterhalt zu bestreiten.*“*; *„*Ich kann/wir können seit Beginn der Corona-Krise eine oder mehrere Forderungen/Rechnungen [z.* *B. Stromrechnung, Kreditrate, Miete usw.] nicht termingerecht bezahlen*“. Zukunftsängste wurden mittels einer 11-teiligen Skala erhoben (0 – gar keine Sorgen bis 10 – sehr große Sorgen): „*Wie viele Sorgen machen Sie sich, dass Sie aufgrund der Corona-Krise finanzielle Probleme bekommen?*“, wobei Werte ab 7 als „große Sorgen“ kodiert wurden. Der Fokus der Analyse ist auf der Messung der subjektiven finanziellen Lage auf der Ebene von Haushalten, unter der Annahme, dass in Familien mit minderjährigen Kindern die Einkommen der Eltern meist zum Wohle aller Haushaltsmitglieder zusammengelegt werden (Hamplova und Bourdais 2009; Ponthieux 2013). Unterschiede in der finanziellen Lage von Frauen und Männern *innerhalb* von Haushalten sind nicht im Fokus der vorliegenden Analyse (Knittler und Heuberger 2018).

## Ergebnisse

### Veränderung der Erwerbssituation

In einem ersten Schritt untersuchten wir, inwieweit Eltern von den Verwerfungen am Arbeitsmarkt betroffen waren, insbesondere in Bezug auf Arbeitslosigkeit und Corona-Kurzarbeit. Die AKCOVID-Befragung zeigt, dass im Juni 2020 bei rund 36 % der Paare mit minderjährigen Kindern zumindest ein Elternteil arbeitslos oder in Kurzarbeit war; in rund 7 % dieser Familien waren gar beide Elternteile entweder arbeitslos oder in Kurzarbeit (Tab. 2). Besonders stark betroffen waren Eltern mit Kindern im Alter von unter sechs Jahren (rund 41 %) sowie Familien mit drei oder mehr Kindern (rund 45 % mit zumindest einem Elternteil im Juni 2020 arbeitslos oder zu Kurzarbeit angemeldet). Unter den Alleinerziehenden waren im Juni 2020 rund 19 % arbeitslos oder in Kurzarbeit. Die Betroffenheit laut Tab. 2 bezog sich dabei vor allem auf Kurzarbeit und weniger auf Arbeitslosigkeit. Bei den Paaren mit minderjährigen Kindern waren rund 20 % der Mütter und rund 22 % der Väter betroffen (rund 5 % der Väter und 9 % der Mütter arbeitslos und rund 12 % der Mütter und 16 % der Väter in Kurzarbeit).

**Table 2:** 

	Von Arbeitslosigkeit oder Kurzarbeit betroffen (in %)			*N*
	Ein Elternteil	Beide Eltern	Gesamt	
*Paare mit zumindest 1 Kind <18 Jahre*	*29,7* *%*	*6,6* *%*	*36,3* *%*	*799*
Paare, jüngstes Kind <6 Jahre	34,7 %	6,5 %	41,2 %	392
Paare, jüngstes Kind 6 < 18 Jahre	26,6 %	6,7 %	33,3 %	407
Paare, 1 Kind <18 Jahre	29,2 %	8,0 %	37,2 %	345
Paare, 2 Kinder <18 Jahre	28,4 %	4,5 %	32,9 %	358
Paare, 3 Kinder <18 Jahre	36,8 %	8,6 %	45,4 %	96
*Alleinerziehende mit zumindest 1 Kind <18 Jahre*	*–*	*–*	*18,6* *%*	*106*

Quelle: AKCOVID-Survey Juni 2020. Sample: Paare und Alleinerziehende mit Kind/ern unter 18 Jahren, gewichtet

Während die Administrativdaten keine Analyse auf der Paarebene erlauben, zeigen die Befragungsdaten mithin eine Kumulation der Einkommensrisiken auf der Ebene von Paaren. Eltern kleiner Kinder zeigten sich aufgrund ihrer Altersstruktur besonders stark betroffen (denn Kurzarbeit betraf vor allem jüngere Personen, cf. Vogtenhuber et al. 2021). Auch die relativ starke Betroffenheit von Mehrkindfamilien liegt im sozioökonomischen Profil dieser Familienkonstellation begründet (häufig niedrig qualifizierte Eltern mit geringen Arbeitsmarktchancen).

Betrachtet man die Erwerbsverläufe von Personen, die im Februar 2020 noch über der Geringfügigkeitsgrenze erwerbstätig waren auf Basis von Administrativdaten, zeigt sich, dass im Juni 2020 rund 16 % der Männer und 14 % der Frauen in Kurzarbeit sowie knapp unter 5 % der Frauen und Männer arbeitslos waren (Steiber et al. 2021b, S. 8), wobei es im Gegensatz zu den Entwicklungen in anderen Ländern in dieser Hinsicht kaum Unterschiede zwischen Frauen ohne Kinder und Müttern gibt (ibid.). Fokussiert man mittels der AKCOVID-Befragungsdaten auf Familien mit minderjährigen Kindern, in denen vor Beginn der COVID-19-Krise *beide* Elternteile über der Geringfügigkeitsgrenze erwerbstätig waren, tritt zutage, dass auch in dieser *erwerbsnahen* Subgruppe in jeder dritten Familie im Juni 2020 zumindest ein Elternteil entweder arbeitslos oder in Kurzarbeit war (Tab. 3). Besonders stark betroffen waren wiederum Eltern von Kleinkindern sowie Mehrkindfamilien.

**Table 3:** 

	Von Arbeitslosigkeit oder Kurzarbeit betroffen (in %)			*N*
	Ein Elternteil	Beide Eltern	Gesamt	
*Paare mit zumindest 1 Kind <18 Jahre*	*25,6* *%*	*8,0* *%*	*33,6* *%*	*493*
Paare, jüngstes Kind <6 Jahre	31,1 %	8,8 %	39,9 %	179
Paare, jüngstes Kind 6 < 18 Jahre	23,4 %	7,8 %	31,2 %	310
Paare, 1 Kind <18 Jahre	28,5 %	9,5 %	38,0 %	216
Paare, 2 Kinder <18 Jahre	21,8 %	5,8 %	27,6 %	226
Paare, 3 Kinder <18 Jahre	[30,0 %]	[11,7 %]	[41,7 %]	[47]
*Alleinerziehende mit zumindest 1 Kind <18 Jahre*	*–*	*–*	*18,5* *%*	*85*

Quelle: AKCOVID-Survey Juni 2020. Sample: Als erwerbsnah gelten Paare mit zumindest einem minderjährigen Kind, in denen beide Elternteile im Februar 2020 über der Geringfügigkeitsgrenze beschäftigt bzw. selbstständig tätig waren. Alleinerziehende gelten als erwerbsnah, wenn sie im Februar 2020 über der Geringfügigkeitsgrenze erwerbstätig oder selbstständig tätig waren.

Die Analysen zeigen mithin, dass es aufgrund der Pandemie in jeder dritten Familie zu einer Veränderung im Erwerbsstatus der Eltern gekommen ist, die in einem direkten Zusammenhang mit Einkommenseinbußen stand. Im Folgenden wird untersucht, wie sich diese Veränderungen auf die finanzielle Lage der Familien ausgewirkt haben.

### Veränderung der finanziellen Lage der Haushalte

**Figure 1:**
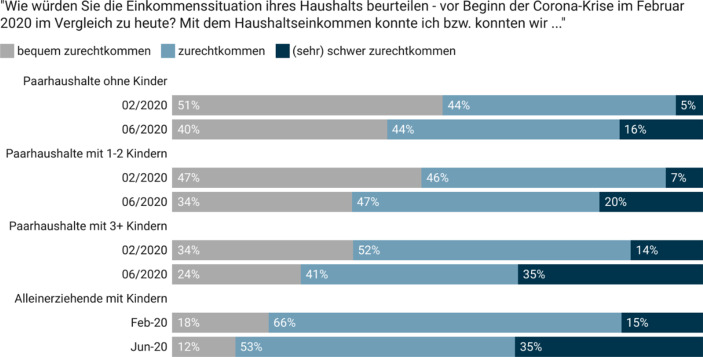

Laut AKCOVID-Befragung verzeichneten unter jenen, die im Juni 2020 unselbstständig erwerbstätig waren, rund 27 % Lohneinbußen – in den meisten Fällen im Kontext der Kurzarbeit. Unter den selbstständigen Erwerbstätigen verzeichneten laut Befragung rund 38 % *starke* Umsatzeinbußen. Daraus folgte, dass im Frühsommer 2020 rund 30 % der Befragten angaben, dass sie im Vergleich zu Februar 2020 mit einem niedrigeren Haushaltseinkommen zurechtkommen mussten (Steiber 2021b). Fokussiert auf Paarhaushalte mit minderjährigen Kindern zeigt sich, dass im Februar 2020 noch etwa 45 % *bequem* mit dem Haushaltseinkommen zurechtkamen, sich dieser Anteil im Juni jedoch auf rund 33 % reduzierte. Umgekehrt erhöhte sich in diesem Zeitraum der Anteil dieser Familien, die nur mehr *(sehr) schwer* mit ihrem Haushaltseinkommen zurechtkamen von rund 8 % auf 21 % (in Abb. 1 werden diese Paare unterteilt in jene mit bis zu zwei Kindern und jene mit drei oder mehr Kindern). Auch unter den kinderlosen Paarhaushalten stieg der Anteil jener, die nur *(sehr) schwer* mit ihrem Haushaltseinkommen zurechtkamen, deutlich (von rund 5 % auf 16 %). Im Vergleich dazu fiel der Anstieg des Anteils subjektiv armutsgefährdeter Familien unter den Alleinerziehenden und Mehrkindfamilien deutlich stärker aus: Unter den Alleinerziehenden erhöhte sich der Anteil jener, die nur mehr *(sehr) schwer* zurechtkamen zwischen Februar und Juni 2020 um 20 Prozentpunkte auf mehr als ein Drittel (35 %). Der Anteil der Alleinerziehenden mit finanziellen Problemen hat sich damit krisenbedingt mehr als verdoppelt (Abb. 1). Zu einer verstärkten subjektiven Armutsgefährdung kam es auch für Familien mit drei oder mehr Kindern: Während im Februar 2020 noch rund 14 % *(sehr) schwer* mit dem Haushaltseinkommen auskamen, belief sich dieser Anteil im Juni 2020 auf rund 35 %.

Dieser Anstieg des Anteils *subjektiv armutsgefährdeter* Familien manifestiert sich für viele Familien als finanzielle Notlage: Rund 19 % der Paare mit minderjährigen Kindern sowie rund 24 % der Alleinerziehenden stimmten im Juni 2020 der Aussage zu, dass sie bereits auf Ersparnisse zurückgreifen oder Schulden machen mussten, um den normalen Lebensunterhalt zu bestreiten. Und rund 9 % der Paare mit Kindern sowie 14 % der Alleinerziehenden konnten ihre Rechnungen (z. B. die Energie- oder Mietkosten) nicht mehr termingerecht bezahlen.

Besonders deutlich sind die Pandemieauswirkungen in „erwerbsnahen“ Paarhaushalten mit Kindern, in denen im Februar 2020 beide Eltern über der Geringfügigkeitsgrenze erwerbstätig waren, im Juni 2020 jedoch mindestens ein Elternteil arbeitslos *oder* für Kurzarbeit angemeldet war. Während vor der Krise noch rund 55 % dieser Familien *bequem* von ihrem Haushaltseinkommen leben konnten, sank dieser Anteil bis Juni 2020 auf unter 30 %. Gleichzeitig stieg der Anteil derer, die nur mehr *(sehr) schwer* zurechtkamen von rund 4 % auf etwa ein Drittel an (keine Abbildung, *N* = 165). Rund 27 % dieser Paare mussten im Juni 2020 auf Ersparnisse zurückgreifen oder Schulden machen, um ihren normalen Lebensunterhalt zu bestreiten, und rund 9 % konnten ihre Rechnungen nicht mehr termingerecht bezahlen.

### Finanzielle Zukunftsängste

**Figure 2:**
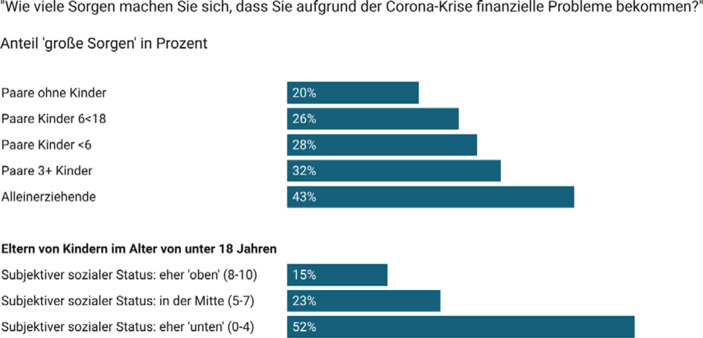

Über die manifesten Auswirkungen der Pandemie auf die finanzielle Lage von Haushalten hinausgehend führten die ökonomischen Folgen auch zu weitverbreiteten Zukunftsängsten: Je nach Alter und Anzahl der Kinder machten sich rund ein Viertel bis ein Drittel der Paare *große Sorgen* (Werte ab 7 auf der Sorgenskala von 0–10), dass sie aufgrund der Corona-Krise in eine finanzielle Schieflage geraten könnten (Abb. 2). Dies traf auf mehr als 30 % der Paare mit drei oder mehr Kindern bzw. auf rund 43 % der Alleinerziehenden zu, während dieser Anteil bei den Paaren ohne Kinder bei rund 20 % lag. Zukunftsängste waren v. a. unter jenen Eltern stark verbreitet, die sich ohnehin schon zu den weniger privilegierten Gruppen der Gesellschaft zählten (MacArthur Skala, cf. Hoebel et al. 2015). Mehr als die Hälfte der Eltern, die ihren eigenen *sozialen Status* auf einer Skala von 0–10 als „eher niedrig“ einschätzten (Werte 0–4), äußerten *große* Sorgen um ihre finanzielle Zukunft, während dies unter den Eltern, die ihre soziale Position als „eher oben“ einschätzten (Werte 8–10), auf rund 15 % zutraf (Abb. 2). Dies ist im Einklang mit Befunden aus dem Austrian Corona Panel Project: im Juni 2020 erwarteten v. a. die Haushalte in den untersten Einkommensdezilen eine Verschlechterung ihrer finanziellen Lage (Albacete et al. 2021, S. 123).

## Fazit und Ausblick

Diese Forschungsnotiz liefert Belege, dass bereits drei Monate nach Beginn der Krise viele Familien in Österreich direkt von den wirtschaftlichen Folgen der COVID-19-Pandemie betroffen waren. Krisenbedingte Kurzarbeit und Arbeitslosigkeit führten zu stark veränderten Erwerbssituationen und finanziellen Einbußen, die nur zu einem Teil durch finanzielle Hilfen abgemildert werden konnten. Fast jedes fünfte Paar mit Kindern unter 18 Jahren und knapp ein Viertel der Alleinerziehenden mussten im Juni 2020 bereits auf Ersparnisse zurückgreifen oder Schulden machen, um den normalen Lebensunterhalt zu bestreiten. Viele Familien gerieten in eine finanzielle Schieflage, insbesondere Alleinerziehende und Mehrkindfamilien (finanziell vulnerable Bevölkerungsgruppen). Der Anteil der Familien mit Kindern, die nur mehr schwer mit dem Haushaltseinkommen auskamen, hat sich zwischen Februar und Juni 2020 mehr als verdoppelt und erreichte bei den Alleinerziehenden und Mehrkindfamilien einen Anteil von mehr als einem Drittel. Viele Menschen sorgten sich im Juni 2020 um ihre finanzielle Zukunft, insbesondere Eltern kleiner oder vieler Kinder bzw. mehr als jede*r vierte Alleinerziehende.

Während Studien aus Großbritannien, Kanada und den USA zeigen, dass Eltern in der COVID-19-Krise häufiger von Arbeitsplatzverlusten und Einkommenseinbußen betroffen waren als Kinderlose (Cheng et al. 2021; Fuller und Qian 2021; Lofton et al. 2021), war dies in Österreich nicht der Fall: Mütter hatten kein höheres Risiko ihren Job zu verlieren oder zu Kurzarbeit angemeldet zu werden als kinderlose Frauen (Steiber et al. 2021a, b auf Basis von Registerdaten). Innerhalb der Familien sehen wir allerdings eine deutliche Variation: Eltern kleiner Kinder waren am häufigsten von Arbeitsplatz- und Einkommenseinbußen betroffen, da jüngere Menschen ein höheres Arbeitslosigkeitsrisiko haben und häufiger zu Corona-Kurzarbeit angemeldet wurden (Steiber et al. 2021a, S. 6).

Die Ergebnisse der Studie zeigen, dass sich die *subjektive Armutsgefährdungsquote* durch die Pandemie in allen Bevölkerungsgruppen erhöht hat und in den vulnerablen Bevölkerungsgruppen, wie den Alleinerziehenden und Mehrkindfamilien, die bereits vor der Pandemie stärker armutsgefährdet waren, besonders stark angestiegen ist. Dementsprechend hatten diese Gruppen und insbesondere Eltern, die sich auf der sozialen Leiter „eher unten“ verorten, die größten finanziellen Zukunftsängste. Gleichzeitig finden wir, dass auch jene Familien, die durch ihre Erwerbsnähe vor Armut geschützt schienen (d. h. zwei erwerbstätige Eltern im Februar 2020), in dieser Krise häufig finanzielle Einbußen verzeichneten.

Ein Vergleich mit den Ergebnissen aus den Simulationsstudien (Fink et al. 2020), die in der Frühphase der Pandemie eine leichte Verringerung der Ungleichheit in der Verteilung von Haushaltseinkommen diagnostizierten, ist schwierig, da die vorliegende Studie auf eine spezifische Subgruppe der privaten Haushalte in Österreich fokussiert – auf Familien mit minderjährigen Kindern, die nur etwa ein Fünftel der Haushalte ausmachen (Statistik Austria 2021b) und in den untersten Dezilen der äquivalisierten Haushaltseinkommen unterrepräsentiert sind. Auch unsere Studie zeigt, dass sich die Krise auf die finanzielle Lage aller Bevölkerungsschichten auswirkte, die finanziell vulnerablen Bevölkerungsgruppen verkrafteten finanzielle Schocks jedoch deutlich schlechter und sind näher an der Schwelle zur Armut.

Auf Basis unserer Analysen konnten lediglich die kurzfristigen Folgen der Pandemie dargestellt werden. Auf Basis weiterer Studien gilt es zu erforschen, welche längerfristigen Folgen die Pandemie für die ökonomische Situation und Armutsgefährdung von Familien haben wird. Es steht zu befürchten, dass sich der Anteil der armutsgefährdeten Familien durch die Krise nachhaltig erhöht hat, insbesondere in vulnerablen Bevölkerungsgruppen. Erste Analysen der zweiten AKCOVID-Befragung zeigen, dass der Anteil der subjektiv armutsgefährdeten Paarhaushalte mit minderjährigen Kindern auch zehn Monate nach Beginn der Krise (Daten für Jänner 2021) auf dem hohen Niveau von rund 21 % verblieben ist (Vorkrisenniveau rund 8 %, cf. Steiber et al. 2021a).

Die vulnerable Situation vieler Familien in Österreich ist unter dem Gesichtspunkt einer nachhaltigen Bekämpfung von Kinderarmut alarmierend. Familien mit Kindern haben häufig wenig finanziellen Spielraum, hohe Fixkosten und im Kontext traditioneller Rollenverteilung oft nur ein Vollzeiteinkommen. Viele Familien können daher finanzielle Einbußen, auch wenn diese temporär sind (z. B. bei der Kurzarbeit), schlecht verkraften und kommen sehr rasch an die Armutsgefährdungsschwelle. Eltern sind in dieser Krise durch die Verwerfungen am Arbeitsmarkt in Kombination mit dem Wegfall von Betreuungsoptionen und den Schulschließungen besonders stark belastet. Besonders bedeutsam sind die Folgen für die Kinder. Das deutsche Jugendinstitut zeigte, dass Kinder aus Familien, die nur mehr schwer mit dem Haushaltseinkommen auskamen, besonders stark von Einsamkeit in Zeiten von Corona betroffen waren (Langmeyer et al. 2020). Auch wirken sich finanzielle Probleme in der Krise direkt auf den Bildungserfolg der Kinder aus (Hupkau et al. 2020) – mit potenziell langfristigen Auswirkungen auf soziale Ungleichheit und intergenerationale soziale Mobilität.

In der vorliegenden Studie, die sich auf die subjektive finanzielle Lage von Familien mit Kindern fokussierte, wurde die Einkommenssituation der Haushalte von Männern und Frauen ähnlich eingeschätzt.^5^ In weiterführenden Studien gilt zu untersuchen, inwiefern sich die Pandemie auf die finanzielle Lage von Männern und Frauen *innerhalb* von Haushalten verändert hat. Durch die Strukturierung von Einkommen innerhalb von Haushalten kann es zu einer stärkeren Armutsgefährdung von Frauen und insbesondere Müttern kommen.

## Footnotes

1 Entscheidend für Anspruch auf und Höhe der Ersatzleistung sind die Dauer und Entlohnung der bisherigen Beschäftigung. Leistungsbezieher*innen erhalten Zuschläge für Kinder mit Anspruch auf Familienbeihilfe und, wenn Anspruch auf einen Zuschlag für ein minderjähriges Kind besteht, auch einen Zuschlag für Partner*innen ohne oder mit einem geringen Einkommen.

2 Als armutsgefährdet gelten Personen, deren *äquivalisiertes Haushaltseinkommen* (Pro-Kopf-Einkommen, das nach der Zahl der Haushaltsmitglieder und dem Alter von Kindern gewichtet wird) weniger als 60 % des nationalen Median-Haushaltseinkommens beträgt. Die Zahlen beziehen sich auf das Jahreseinkommen aus dem Kalenderjahr 2019.

3 Im Rahmen der EU-SILC Berichterstattung: Der EU-SILC Tabellenband 2021, der im Frühjahr 2022 publiziert wird, wird erstmals registerbasierte Jahreseinkommen für das Jahr 2020 beinhalten.

4 Der Interviewzeitraum spannte sich vom 18. Juni bis zum 2. Juli 2020. Da mehr als 90 % der Interviews im Juni stattfanden, wird der Erhebungszeitpunkt in den Tabellen und Grafiken der Einfachheit halber mit Juni 2020 angegeben.

5 Regressionsanalysen zeigen, dass es keinen Unterschied macht, ob Frauen oder Männer stellvertretend für den gesamten Haushalt eine Einschätzung bzgl. der finanziellen Lage des Haushalts treffen.
